# Vehicle operating speeds in southwestern Colombia: An important database for the future implementation of optimization models for geometric design of roads in mountain topography

**DOI:** 10.1016/j.dib.2020.106210

**Published:** 2020-08-22

**Authors:** Fernando Jove Wilches, Jorge Luis Argoty Burbano, Edilberto Elías Contreras Sierra

**Affiliations:** a Department of Civil Engineering, Universidad de Sucre, Sincelejo, Sucre, Colombia; b Department of Civil Engineering, Universidad de Nariño, San Juan de Pasto, Nariño, Colombia; c Universidad de Sucre, Sincelejo, Sucre, Colombia

**Keywords:** Operating Speed, Traffic speed, Design speed, Spot speed, Geometric design of roads, Speed profiles

## Abstract

The essential objective of a road is framed in allowing the circulation of vehicles from a point of origin to a destination, being essential to fulfil completely this function, consider aspects such as: functionality, safety, economy, comfort, environmental integration and harmony or aesthetics. For this effect, a geometric design based on consistency must be achieved, which arranges the elements of the road and their geometric characteristics, helping to minimize adverse effects on the driver's expectations, perceiving homogeneity on the route, without abrupt variation in the level of attention necessary and thus be able to adapt to the changing geometric conditions of the road. In order to assess the consistency in the geometric design, different criteria and methodologies strongly related to the level of accident rates have been developed, which in turn are based on the analysis of the evolutions of the operating speeds. By virtue of the above, speed is therefore of vital importance to carry out any type of evaluation or analysis of traffic, since it is an important indicator of the quality of the service offered to users, because of it is function of the physical characteristics of the road and its environment. Among the main characteristics of vehicular traffic that can be studied from speed data, we have: the intensity of circulation, vehicle speeds and travel time, origin and destination of trips, vehicular accidents, among others. The purpose of this document is to present a database of speed obtained on a road located in the Pasto (Nariño department) in southwestern Colombia. The analysed road sector has a total length of 27.5 km and is developed in a predominantly mountainous topography. The data collected corresponds to the geometric characteristics of the road, as well as the design, traffic and operating speeds of each element throughout the sector. The data series corresponds to 312 elements of the geometric design, which are located continuously along sector. The design and road speed for each element, was determined from the geometric characteristics of the road; meanwhile, the operating speed was determined using spot speed data, which was obtained from field measurements with the Bushnell Radar Gun. For the determination of the Operating Speed in each element of the layout and for each class of vehicle considered (cars, buses and two-axle trucks), in each direction of movement, a minimum sample size of 64 Spot Speed data was used. The speed dataset is of great importance, because it provides traffic and transport engineering with relevant information for investigations such as: analysis of traffic accidents, establishment of design elements, traffic operation plans, regulation and control, zones with speed problems, study of traffic flows and finally, the assignment of design speeds for similar and future projects.

**Specifications Table**

**Table:** 

**Subject**	Civil and Structural Engineering
**Specific subject area**	Roads and Transportation Engineering
**Type of data**	Table Chart
**How data were acquired**	Collection of base data through road design plans and memories, determination of road and design speeds, field data collection of spot speeds, calculation of operating speeds and generation of speed profiles, Microsoft Excel 2013.
**Data format**	Raw Analysed
**Parameters for data collection**	The field data were collected in each of the elements of the road sector analyzed (K5+000 - K32+500). Field data were collected on weekdays, except holidays and under favorable weather conditions; the pavement surface being in good passable conditions throughout the evaluated sector and under free-flow conditions, during non-peak times.
**Description of data collection**	Estimation of design, road and operating speed data of a road sector, through statistical calculations and spot speed measurements on the road, along 312 continuous elements (tangents and curves) belonging to the road sector analyzed.
**Data source location**	Institución: Universidad de Nariño City: San Juan de Pasto Region: Nariño Country: Colombia
**Data accessibility**	Raw and analysed data was deposited in the Mendeley repository as Data, v1, 2020. DOI: 10.17632/6n2pwnhh66.1http://dx.doi.org/10.17632/6n2pwnhh66.1

**Value of the Data**

• The data in this article can be used to: a) determine critical sites for the occurrence of traffic accidents, b) verify the consistency in the geometric design and identify elements of the road layout that require adjustments and c) identify homogeneous sections along the road layout.
• The speed data is useful for researchers, institutions, and specialists in the subject, who seek the implementation of models to optimize the geometric design of roads, in countries with topographic and socioeconomic characteristics similar to those of Colombia.
• The dataset of speed can be used to carry out the analysis of capacity and service levels, in order to measure the quality of vehicular traffic flow, within a road sector that develops in mountainous topography.
• The dataset provides inputs necessary for estimating vehicle operating costs for automobiles, buses, and trucks.
• The data can provide those responsible for the road, the decision-making for the implementation of speed regulation devices and complementary signalling, in order to improve the safety and comfort of road users.

## 1 Data description

**Fig 1:**
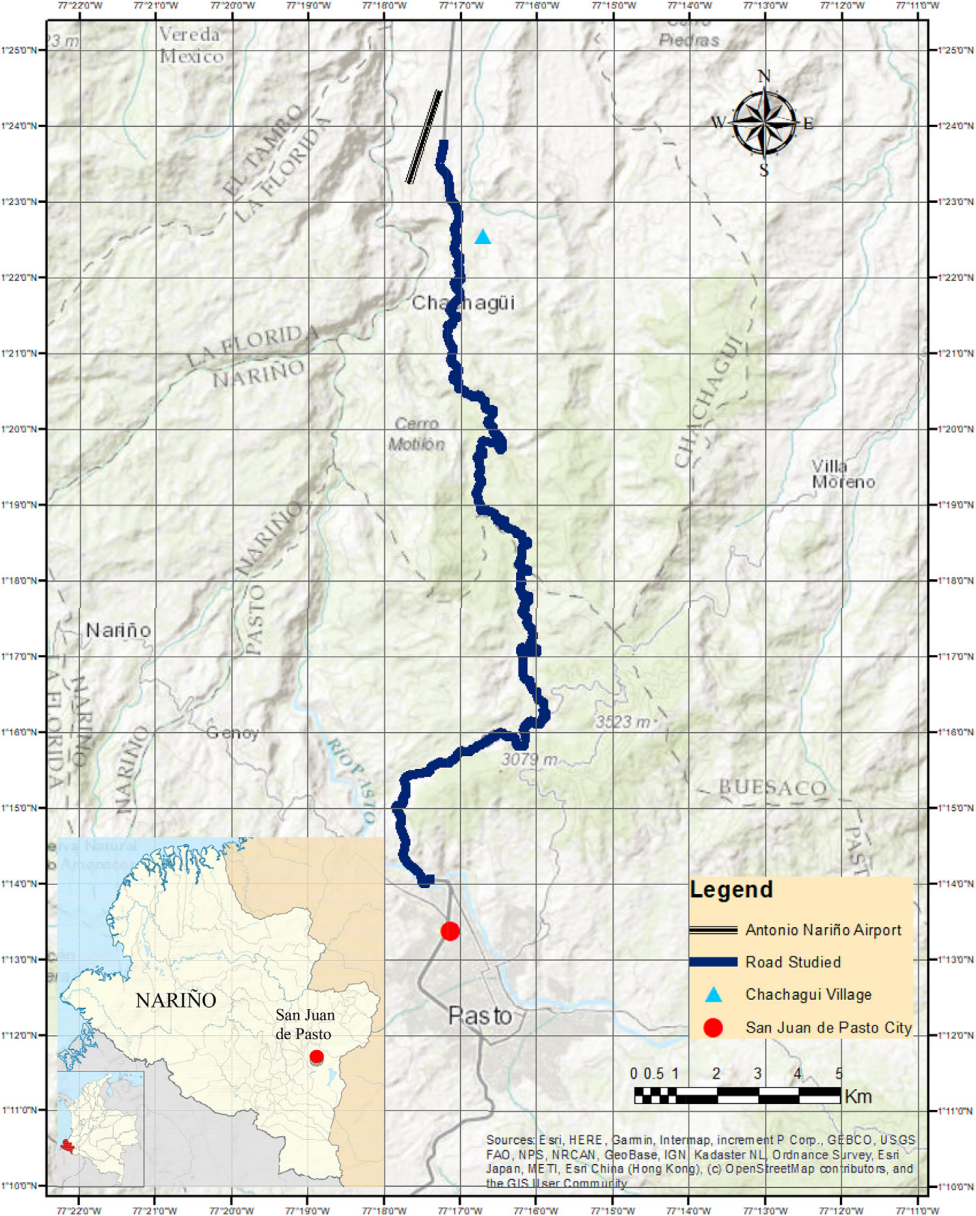
Location of the San Juan de Pasto - Chachagui road in the department of Nariño (Colombia).

**Fig. 2:**
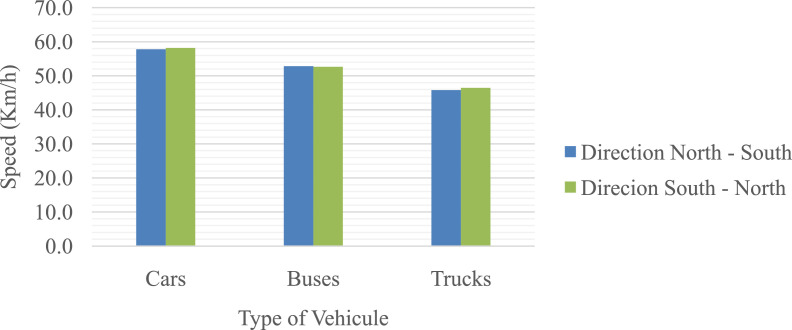
Average operating speeds for the entire road sector studied.

**Fig. 3:**
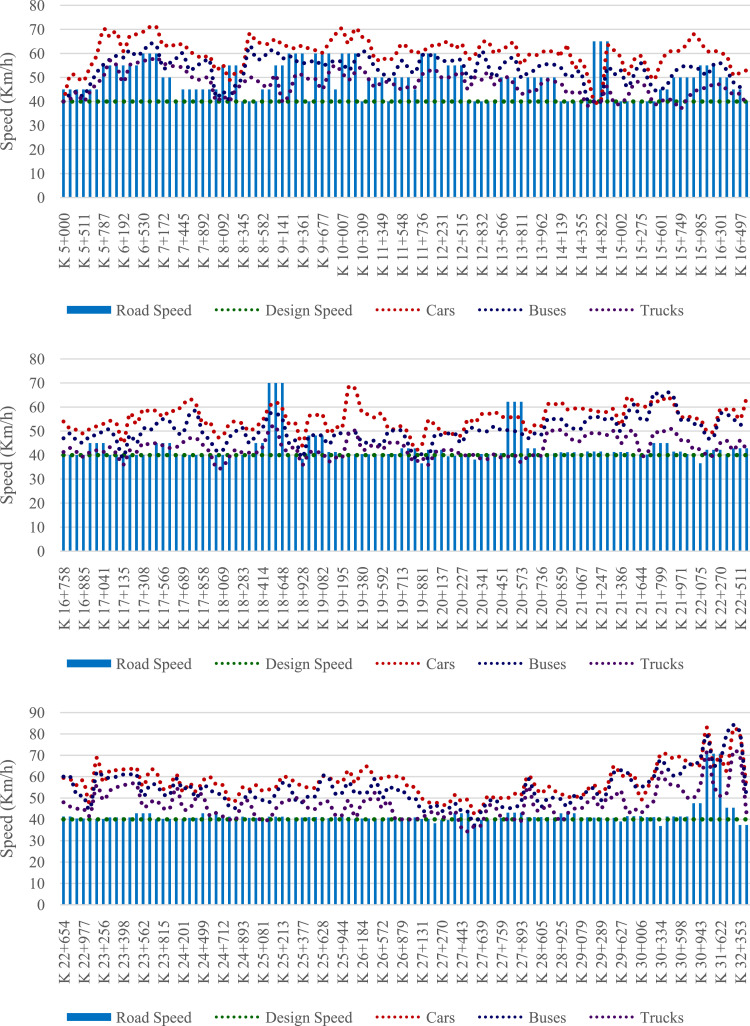
Speed data of the evaluated sector (K5+000 - K32+500), North - South direction.

**Fig. 4:**
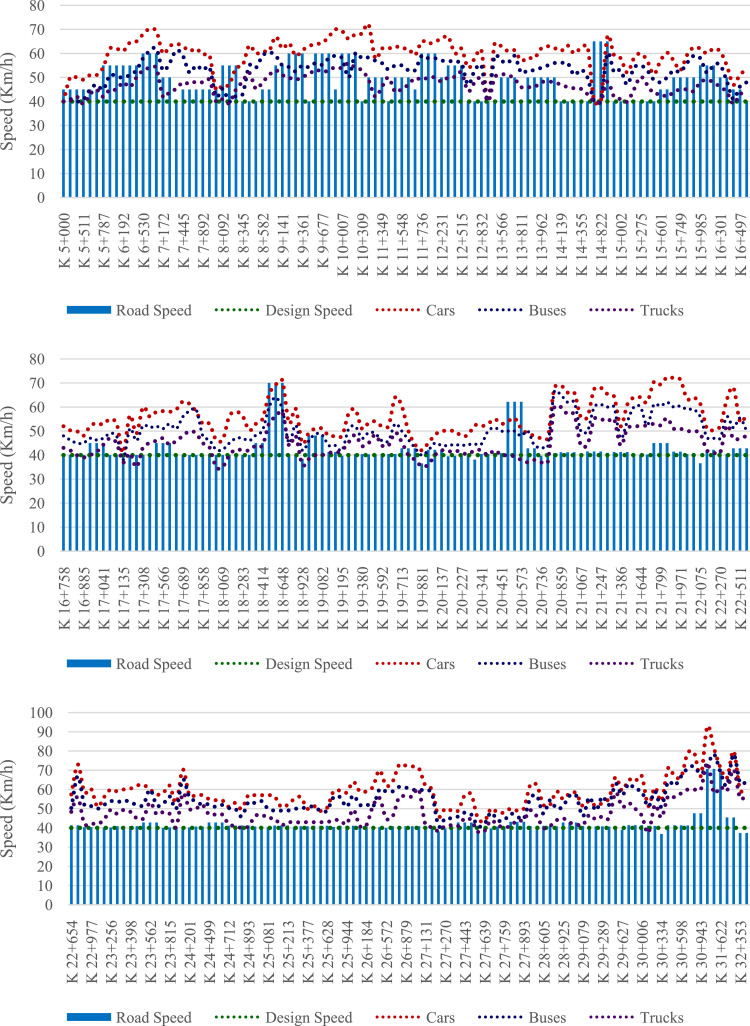
Speed data of the evaluated sector (K5+000 - K32+500), South - North direction.

Tables and figures with the design, road and operating speeds were obtained, analysed and processed based on the geometric design data of the studied road and from the data obtained from field measurements on 312 elements of the located geometric layout on a road that connects San Juan de Pasto city with Chachagui town, located in the department of Nariño (Colombia). This section will show the main data collected and processed for the determined speeds. The raw and processed data files were deposited in the Mendeley data repository DOI: 10.17632/6n2pwnhh66.1 http://dx.doi.org/10.17632/6n2pwnhh66.1. Fig. 1 shows the location of the road studied. There you can see the 27.5 km of road that connect the two urban areas mentioned. Table 1 shows the average operating speeds calculated for approximately each kilometre of the journey, for each class of vehicle considered and for each direction of movement. Fig. 2 shows the average operating speed of the entire road sector for Cars, Buses and C2 trucks, for each road direction. In Fig. 3, Fig. 4, the speed data found for the North-South and South-North directions of circulation are shown for each of the elements evaluated.

**Table 1:** Average operating speeds calculated for each kilometer of the road.

ROAD SECTOR	INITIAL STATION	FINAL STATION	AVERAGE OPERATING SPEED (Km/h)			AVERAGE OPERATING SPEED (Km/h)		
			DIRECTION NORTH - SOUTH			DIRECTION SOUTH - NORTH		
			Cars	Buses	Trucks	Cars	Buses	Trucks
1	KM 5+000	KM 6+042	55.1	47.6	45.4	50.9	43.7	42.0
2	KM 6+042	KM 7+172	68.0	60.8	55.2	65.7	55.0	50.0
3	KM 7+172	KM 8+021	61.5	56.2	52.3	61.5	55.5	46.5
4	KM 8+021	KM 9+141	59.0	52.6	45.8	56.0	50.0	46.3
5	KM 9+141	KM 10+007	63.1	56.8	48.4	63.9	55.5	51.6
6	KM 10+007	KM 11+259	67.4	57.3	51.3	68.7	56.9	53.2
7	KM 11+259	KM 12+094	60.1	54.3	47.7	61.9	55.5	47.0
8	KM 12+094	KM 13+043	62.5	56.3	50.0	61.7	54.9	49.1
9	KM 13+043	KM 14+027	60.9	53.9	47.1	58.3	53.7	47.5
10	KM 14+027	KM 15+002	55.5	49.5	43.6	58.3	51.8	44.9
11	KM 15+002	KM 16+080	58.8	50.9	42.2	57.5	52.9	44.5
12	KM 16+080	KM 17+041	54.2	49.1	42.9	53.4	48.0	42.9
13	KM 17+041	KM 18+005	56.2	50.1	43.2	55.6	50.4	44.5
14	KM 18+005	KM 19+016	53.1	48.3	41.8	56.1	49.3	44.3
15	KM 19+016	KM 20+034	54.2	46.2	42.0	51.6	46.7	42.4
16	KM 20+034	KM 21+003	54.5	50.2	41.5	52.4	48.3	42.0
17	KM 21+003	KM 22+055	59.6	56.7	47.5	64.8	58.0	51.9
18	KM 22+055	KM 23+016	56.8	53.6	43.8	59.2	53.1	46.6
19	KM 23+016	KM 24+201	60.1	56.8	50.9	59.4	54.1	47.5
20	KM 24+201	KM 25+044	54.6	51.4	44.9	54.8	51.8	45.7
21	KM 25+044	KM 26+053	56.8	52.5	45.1	54.4	51.0	43.5
22	KM 26+053	KM 27+039	60.1	53.7	45.3	65.7	57.3	49.9
23	KM 27+039	KM 28+201	49.2	45.5	40.7	52.0	47.4	42.6
24	KM 28+201	KM 29+006	54.9	51.1	45.6	57.1	52.6	44.2
25	KM 29+006	KM 30+006	56.1	54.5	47.1	57.8	56.0	47.3
26	KM 30+006	KM 31+017	64.2	62.6	52.4	66.3	62.4	53.2
27	KM 31+017	KM 32+500	71.1	72.3	59.2	73.1	69.7	62.4

## 2 Experimental design, materials and methods

### 2.1 Study area description

San Juan de Pasto is the capital of the department of Nariño (Fig. 1), located in the extreme south-west of Colombia, it limits to the north with the department of Cauca, to the east with the department of Putumayo, to the south with Ecuador, and to the west with the Pacific Ocean. The city rises at the foot of the Galeras volcano, has a height of 2559 m above sea level m.a.s.l. and an average annual temperature of 14°C [1].

The Pasto - Chachagui road corridor is part of National Route 25 (or commonly called Troncal de Occidente), which develops from the Rumichaca Bridge on the border with Ecuador and ends in the city of Barranquilla, north of the country. It is the main Roadway of the West of the Country and until now, the only Way that borders the Pacific [2]. The area of interest is 90% developed in mountainous terrain and the remaining 10% in undulating and flat terrain. The road sector mentioned is of particular interest, since it is the road that connects the department of Nariño with the rest of the country's regions, as well as Colombia, with the rest of the countries of South America [3].

### 2.2 Material and methods

A total of 312 consecutive elements (tangents and curves) were selected, corresponding to the geometric design of sector K5+000 - K32+500, which is part of the road known as Route 25-02, on the San Juan de Pasto - Chachagüi road, located in the department of Nariño. The characteristics of the geometric design such as: length of the elements (lengths of inter-tangencies and curves), deflection angles and radius of the horizontal curves, longitudinal slopes and widths of the road were obtained from existing planes and some elements, such as the cross slope (cant), were determined in the field through direct measurement using the Topographic Abney level [4].

Taking into account that the design speeds of the road were not available, it was necessary, based on the radius of the curves and their maximum cant values (measured in the field), to determine these speeds as road speeds of each element. Based on the road speeds of the curves, the road speed on the tangent lines was determined, taking the highest road speed between the curves adjacent to the evaluated tangent.

To determine the types of vehicles to be analyzed, the vehicle categories with the highest incidence and representativeness in terms of operating speed were used, which correspond to: Automobiles, Buses and Two-axle Trucks (C2); since according to the data provided by the Institute Nacional de Vías (INVIAS) [5], the vehicle volumes for trucks of categories C3, C4, C5 and C6 are low, intermittent and develop low speeds, which do not exceed speeds design, and especially when they are loaded.

For the determination of the Operating Speed [6] in each element of the layout and for each class of vehicle considered, in each road direction, it was worked with a minimum sample size of 64 Spot Speed data [7], from according to the equation: N = (K * S / E)2 [8]. To obtain each spot speed, the automatic method determined by the Bushnell Radar Gun was selected, capable of taking readings in 0.25 s [9]. For the selection of the site where the Spot Speed measurements of each element were developed, the following criteria were taken into consideration: in the case of horizontal curves, it was taken in the middle of their length; in the case of tangents, it was taken in the middle of the element, for lengths less than 200 meters and in the last third of the length of the tangent (in the direction of vehicular flow), for lengths greater than 200 meters. Measurements were made on ordinary days, except Sundays, holidays and under favorable weather conditions; the surface of the pavement was in good passable conditions throughout the study sector and under free-flow conditions, during non-peak times.

For the calculation of the operating speed of each class of vehicle, in each element and in each direction of movement, the statistical analysis of the data collected in the field was carried out, using class intervals, to identify the relative and accumulated frequencies, in order to obtain the 85th percentile of the velocity distribution [10]. Table 1 shows the operating speed values for road sections of approximately 1 km in length, for better compression and analysis of the data, this process was performed using the Excel spreadsheet tool.

Finally, figures were prepared to show the speed profiles (Figs. 3 and 4), in which the operating speeds for each category of vehicle, the design speeds of the road section and the road speeds for each element of the route are graphically represented by a dispersion diagram, in the direction of advance of the road, both for the direction of circulation North - South, and for the direction South - North.

## Declaration of Competing Interest

The authors declare that they have no known competing financial interests or personal relationships that could have appeared to influence the work reported in this paper.

## Appendix Supplementary materials

Image, application 1

Image, application 2
